# Clinical Characteristics and Treatment Outcomes of Pediatric Glaucoma: A Retrospective Cohort Study

**DOI:** 10.3390/jcm15093551

**Published:** 2026-05-06

**Authors:** Nancy N. Hanna, Doris Canes Napoles, Aaron Flickinger, Carter L. Carlos, Richard W. Hertle, Xiaoming Gong

**Affiliations:** 1 Vision Center, Akron Children’s Hospital, Akron, OH 44308, USA; nhanna@akronchildrens.org (N.N.H.); dcanesnapoles@akronchildrens.org (D.C.N.); aflickinger@akronchildrens.org (A.F.); ccarlos@akronchildrens.org (C.L.C.); rwhertle@gmail.com (R.W.H.); 2 Rebecca D. Considine Research Institute, Akron Children’s Hospital, Akron, OH 44308, USA

**Keywords:** pediatric glaucoma, secondary glaucoma, cataract surgery, visual outcomes, retrospective cohort

## Abstract

**Objectives**: This study aimed to evaluate clinical characteristics and treatment outcomes in a cohort of pediatric patients with glaucoma over 10-year period at a tertiary referral center. **Methods**: Medical records of patients younger than 18 years diagnosed with glaucoma between 1 January 2013 and 31 December 2023 were retrospectively reviewed. Diagnoses were classified according to the Childhood Glaucoma Research Network (CGRN). Demographic data, clinical characteristics and surgical outcomes were analyzed. Statistical analyses included Student’s *t*-test, one-way ANOVA, and corresponding non-parametric methods (α = 0.05). **Results**: A total of 105 patients (168 eyes) were included, with a mean age of 5.52 ± 5.61 years, and a mean duration of follow-up of 5.41 ± 3.37 years. Mean baseline intraocular pressure (IOP) was 24.41 ± 10.85 mmHg. Secondary glaucoma was the predominant category (69%), led by glaucoma following cataract surgery (26%). Bilateral disease occurred in 60% of cases, more frequently in secondary forms. Surgery was performed in 57.4% of glaucomatous eyes. All subtypes except glaucoma following cataract surgery (GFCS) achieved significant reductions in IOP from baseline (*p* < 0.01). Despite effective IOP control, final visual acuity remained limited in many patients, especially those with glaucoma associated with non-acquired ocular anomalies or following cataract surgery. Worse baseline vision and higher presenting IOP were associated with poorer final acuity. **Conclusions**: Secondary glaucoma, particularly GFCS, was the most common form of pediatric glaucoma in this cohort. Although IOP control was generally successful, visual outcomes frequently remained suboptimal, highlighting the importance of early detection, comprehensive management, and close long-term monitoring.

## 1. Introduction

Childhood glaucoma is a heterogeneous group of vision-threatening disorders with an incidence ranging from 2.29 to 5.41 per 100,000 children [1,2]. It is characterized by elevated intraocular pressure (IOP), which can lead to optic nerve damage and irreversible vision loss due to progressive optic neuropathy, corneal opacity or amblyopia if not promptly diagnosed and managed [3,4]. Early diagnosis and appropriate treatment are crucial for preserving vision and improving quality of life in affected children. Compared to adult-onset glaucoma, childhood glaucoma presents unique diagnostic and management challenges due to its variable clinical presentation, diverse etiologies, and the complexities of managing glaucoma in a developing visual system [5,6].

The establishment of the Childhood Glaucoma Research Network (CGRN) classification has improved consistency across studies by clearly distinguishing primary childhood glaucoma from secondary forms associated with ocular anomalies, systemic syndromes, acquired conditions, or prior ocular surgery [7,8]. Understanding the distribution and behavior of these subtypes is essential, as their prevalence, clinical trajectory, and response to treatment can differ substantially.

Management strategies for pediatric glaucoma typically include both surgical and medical approaches aimed at lowering IOP—the only modifiable risk factor known to influence disease progression. Achieving good outcomes in patients with childhood glaucoma may be challenging for clinicians because of the complex nature of the disease [9]. Despite improvements in surgical techniques and pharmacologic options, many children continue to experience suboptimal long-term visual outcomes, underscoring the need for ongoing research into more effective management approaches. Prior studies have highlighted the benefits of early detection and timely intervention in improving long-term outcomes for children with glaucoma [10,11,12,13,14]. However, comprehensive longitudinal data remain limited, particularly across diverse patient populations, making it essential to evaluate long-term trends and identify factors associated with successful disease control and visual preservation.

This 10-year retrospective cohort study provides an in-depth evaluation of the clinical characteristics, management strategies, and outcomes of pediatric patients with glaucoma treated at a tertiary care center. By analyzing a decade of real-world clinical data, we aim to clarify the distribution of glaucoma subtypes, assess the effectiveness of various treatment approaches, and identify factors associated with visual and IOP outcomes. These findings contribute to the growing evidence base needed to optimize care and improve long-term visual prognoses for children affected by glaucoma.

## 2. Materials and Methods

### 2.1. Study Population and Ethics

This retrospective cohort study included all patients diagnosed with and treated for pediatric glaucoma at a tertiary care, quaternary referral children’s hospital in the United States, Akron Children’s Hospital (Akron, OH, USA), between 1 January 2013, and 31 December 2023. Eligible patients were younger than 18 years at their initial visit and met the CGRN diagnostic criteria for glaucoma or glaucoma suspect. The study protocol was submitted to the Akron Children’s Hospital Institutional Review Board (IRB) for approval. The requirement for written informed consent was waived due to the retrospective nature of the study. The study was compliant with the US Health Insurance Portability and Accountability Act (HIPAA) of 1996 and adhered to the tenets of the Declaration of Helsinki.

### 2.2. Patient Characteristics and Classification

Baseline characteristics included age at presentation, sex and race. Clinical characteristics of each of the patients were evaluated according to the criteria proposed by the CGRN classification system. Patients diagnosed with glaucoma were further classified into three groups: (1) primary childhood glaucoma, including primary congenital glaucoma (PCG) and juvenile open-angle glaucoma (JOAG); (2) secondary childhood glaucoma, including glaucoma associated with acquired conditions (S_GAC; e.g., steroid-induced, uveitis, trauma, retinopathy of prematurity); glaucoma associated with non-acquired ocular anomalies (S_GNOA; e.g., Peter’s anomaly, anterior segment dysgenesis, aniridia, microphthalmia, and angle-closure glaucoma); glaucoma associated with non-acquired systemic/syndromic disease (S_GNSD; e.g., Sturge–Weber syndrome, Walker–Warburg syndrome, Al-Gazali syndrome, Stickler syndrome); and glaucoma following cataract surgery (S_GFCS); (3) glaucoma suspect (GS), defined per CGRN criteria as open anterior chamber angle on gonioscopy, with ≥1 of the following clinical findings: (1) appearance of the optic disk or retinal nerve fiber layer (RNFL) that is suspicious for glaucomatous damage, (2) a visual field suspicious for glaucomatous damage, or (3) consistent elevation of IOP associated with normal appearance of the optic disk and RNFL and with normal visual field test results.

### 2.3. Data Collection

Clinical data extracted from the medical record included IOP, visual acuity (VA), refraction and cup–disk ratio (CDR) at baseline and throughout follow-up. IOP was measured in all patients using a combination of at least two instruments—Goldman or Perkins applanation tonometry (Haag-Streit, SCHWEIZ, Köniz, Switzerland), Tonopen^®^ (Ametek Reichert, Buffalo, NY, USA) pneumotonometry, or Icare^®^ (Icare USA, Inc., Raleigh, NC, USA)—either in the clinical or during anesthetic induction. The VA values were recorded under binocular and monocular conditions using the E-HOTV PEDIG protocol and then converted to the logarithm of the minimum angle of resolution (logMAR) analysis when quantifiable [15,16]. The fixation categories such as a central, steady, and maintained (CSM) were used for patients who were too young to determine the pictures or numbers [17]. The qualitative VA, including count finger (CF), hand motion (HM), light perception (LP), and o light perception (NLP), were noted when standard testing was not feasible. Refractive errors were documented as spherical equivalent.

Interventions during the follow-up course and final outcomes (VA and IOP) at the latest available visit in eyes with a confirmed glaucoma diagnosis were recorded. Data for surgical procedures was reviewed to determine if the patients had received any incisional surgeries (e.g., trabeculectomy, trabeculotomy, goniotomy, Baerveldt glaucoma implantation), cyclodestructive laser procedures (e.g., diode laser endoscopic cyclophotocoagulation), or both.

### 2.4. Outcome Measures

The primary outcome measures were distribution of glaucoma subtypes according to CGRN criteria and baseline demographic and clinical characteristics. The secondary outcome measures were management strategies (medical, surgical, or both) and treatment outcomes (IOP and VA) at the final follow-up.

### 2.5. Statistical Analysis

Continuous data were reported as numbers, percentages, and mean ± standard deviation or median ± interquartile range (IQR), where appropriate. The Shapiro–Wilk test was used to assess for the normality of continuous data distribution. Two-tailed Student’s *t*-test was used for comparison between two groups, while one-way ANOVA followed by Tukey’s multiple comparison post hoc tests were used among multiple groups. The Mann–Whitney U test or Kruskal–Wallis test was performed in case of non-normal distribution. All data analyses were performed in R 4.5.1 (The R Foundation). Results were considered statistically significant when a *p*-value of <0.05 was observed.

## 3. Results

### 3.1. Study Population Characteristics

The demographic features and patient characteristics of the study population are presented in Table 1. A total of 105 pediatric patients (168 eyes) were included in the study. Among them, 94 patients (148 eyes) were diagnosed with glaucoma, while 11 patients (20 eyes) were classified as glaucoma suspects. The cohort was predominantly Caucasian (68.57%). Gender distribution was not significantly different across groups (*p* = 0.748), although a slight male predominance was observed overall (male-to-female ratio: 1.14:1). Subgroup analysis revealed a male predominance in S_GAC (male-to-female ratio: 3.2:1) and a female predominance in S_GNOA (male-to-female ratio: 1:2.5).

The mean age at presentation was 5.52 ± 5.61 years (median 1.58; IQR, 0.25–6.75), and the mean follow-up duration was 5.41 ± 3.37 years (median 5.0; IQR, 2.2–11.2). Bilateral disease was present in 60% of patients with no significant difference across subtypes (*p* = 0.481). JOAG (94.7%), S_GNSD (68.8%), S_GFCS (62.5%), and GS (81.8%) were associated with bilateral disease, whereas PCG and S_GAC were predominantly unilateral (71.4% and 71.2%, respectively).

Of all glaucomatous eyes, 31% were primary and 69% were secondary. JOAG accounted for 37 eyes (22%) and PCG for nine eyes (6%). Among the secondary glaucoma, the most prevalent subtype was S_GFCS, affecting 39 eyes (26%), including 23 aphakic eyes, seven pseudophakic eyes, and nine eyes with congenital cataract associated with ocular anomalies or systemic disease. Most patients (92%) had open-angle glaucoma (>50% open), while 8% had angle-closure glaucoma (<50% open or acute angle closure). Among the 26 S_GAC eyes (18%), open angles were observed in 90.5% and closed angles in 9.5%. Underlying causes included trauma (33.4%), steroid-induced glaucoma (28.6%), and retinopathy of prematurity (8.3%). S_GNSD accounted for 27 eyes (18%), most commonly due to phakomatoses such as Sturge–Weber syndrome, Von Hippel–Lindau disease, and neurofibromatosis. Other syndromes included Al-Gazali syndrome and Stickler syndrome. S_GNOA affected 10 eyes (6%), predominately associated with aniridia, Peter’s anomaly, and unspecified anterior segment dysgenesis.

### 3.2. Baseline Ocular Characteristics

Baseline ocular characteristics are summarized in Table 2. At presentation, primary glaucomas (PCG and JOAG) had significantly larger cup-to-disk ratios (CDRs) compared with secondary glaucomas (*p* = 0.01), indicating more advanced optic nerve cupping at diagnosis. Within secondary glaucoma subtypes, eyes with S_GNOA exhibited significantly smaller CDRs than those with S_GAC (*p* < 0.008), S_GNSD (*p* < 0.001), and S_GFCS (*p* = 0.007). Refractive status also differed across glaucoma subtypes. Myopia was more common in PCG and JOAG, whereas eyes with S_GFCS demonstrated a predominantly hyperopic refractive profile. This pattern is consistent with aphakia, pseudophakia, and altered ocular growth following early cataract extraction.

**Figure 1:**
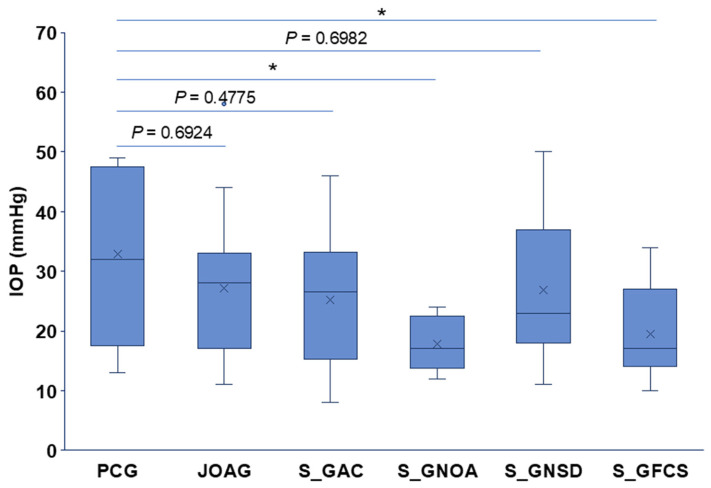
Baseline IOP across glaucoma subtypes. Statistical differences were determined using one-way ANOVA with Tukey post hoc comparisons. Significance levels: * *p* ≤ 0.05. Box plot elements: mean = x, centerline = median, box limits = 25th and 75th percentiles, whiskers = min and max.

The mean baseline IOP across all glaucomatous eyes was 24.41 ± 10.89 mmHg. IOP differed significantly among glaucoma subtypes, with PCG presenting with the highest mean IOP. Specifically, PCG eyes had significantly higher baseline IOP compared with S_GNOA (*p* = 0.028) and S_GFCS (*p* = 0.020), as shown in Figure 1.

Baseline VA was assessed where feasible. As shown in Table 3, quantitative VA measurements were unavailable in 23 patients who were too young for standard testing, though qualitative assessments (e.g., CF, HM, LP, NLP) were recorded. In unilateral glaucoma, non-glaucomatous fellow eyes had substantially better baseline VA than glaucomatous eyes, with logMAR ≤ 0.3 observed in 51.9% vs. 21.7%, respectively. When evaluating vision according to VA category rather than glaucoma status, only 26.3% of eyes with logMAR ≤ 0.3 were glaucomatous, whereas 85.7% of eyes categorized as CF or HM were glaucomatous. Among patients with bilateral glaucoma, 32.9% of eyes exhibited logMAR VA ≤ 0.3 at presentation.

**Figure 2:**
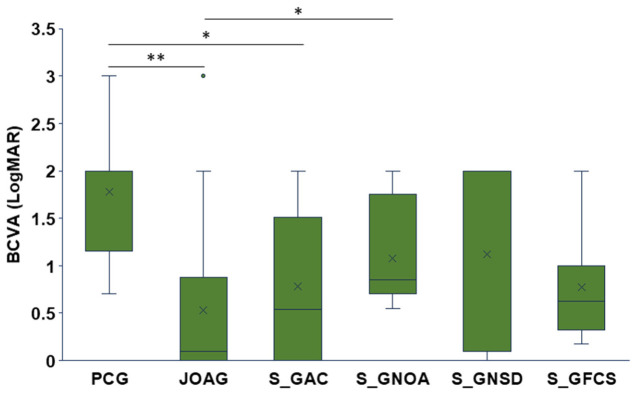
Baseline BCVA (logMAR) for each glaucoma subtype. Differences were evaluated using one-way ANOVA with Tukey post hoc testing. Significance levels: * *p* ≤ 0.05, ** *p* ≤0.01. Box plot elements: mean = x, centerline = median, box limits = 25th and 75th percentiles, whiskers = min and max.

Subtype-specific comparisons revealed that JOAG eyes had significantly better baseline VA than PCG eyes (*p* = 0.0017). Among secondary glaucomas, S_GAC eyes demonstrated superior baseline VA compared with PCG (*p* = 0.033), whereas S_GNOA eyes showed significantly worse baseline VA relative to JOAG (*p* = 0.044) (Figure 2).

### 3.3. Surgical Interventions

Overall, 59 eyes (40%) underwent incisional glaucoma surgery, seven eyes (4.7%) underwent cyclodestructive procedures, and 19 eyes (12.8%) received both at some point during follow-up. Additionally, 63 eyes (42.5%) underwent nonglaucoma procedures during the follow-up.

**Figure 3:**
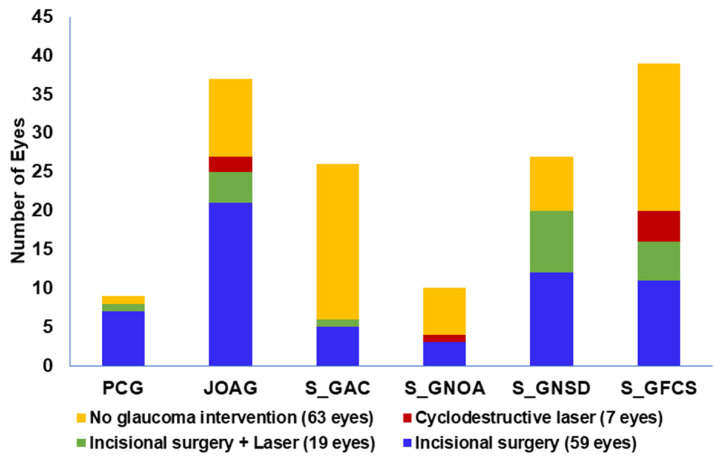
Frequency and type of glaucoma surgical interventions (incisional, cyclodestructive, or combined) performed in each glaucoma subtype during the study period.

The frequency of glaucoma intervention in each subtype is shown in Figure 3, and specific surgical procedures are detailed in Table 4. Primary glaucomas (PCG and JOAG) had the highest surgical burden, whereas surgical intervention in S_GAC was more selectively pursued based on the likelihood of meaningful visual benefit.

### 3.4. Follow-Up Outcomes

**Figure 4:**
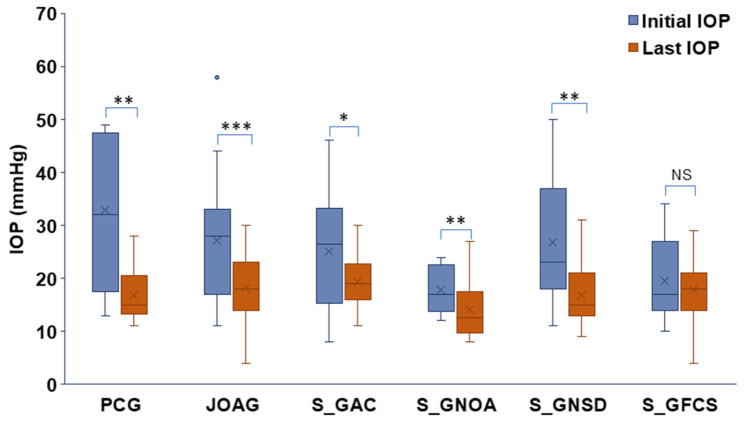
Comparison of IOP at presentation and at the final follow-up visit across glaucoma subtypes. Paired differences were evaluated using two-tailed Student’s *t*-tests. Significance levels: * *p* ≤ 0.05, ** *p* ≤ 0.01, *** *p* ≤ 0.001. Box plot elements: mean = x, centerline = median, box limits = 25th and 75th percentiles, whiskers = min and max.

The mean final IOP across glaucomatous eyes was 17.2 ± 5.4 mmHg. Significant IOP reductions from baseline achieved in PCG (*p* < 0.01), JOAG (*p* < 0.001), S_GAC (*p* < 0.05), S_GNOA (*p* < 0.01), and S_GNSD (*p* < 0.01). Although IOP decreased in S_GFCS, the change did not reach statistical significance (Figure 4).

**Figure 5:**
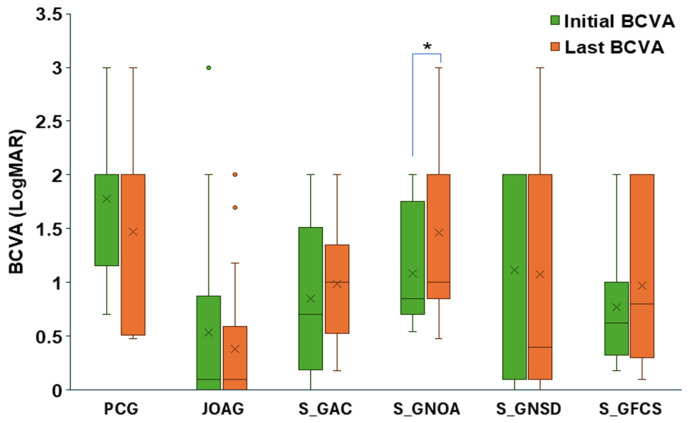
Comparison of BCVA (logMAR) at presentation and at final follow-up for each glaucoma subtype. Differences were assessed using two-tailed Student’s *t*-tests. Significance levels: * *p* ≤ 0.05. Box plot elements: mean = x, centerline = median, box limits = 25th and 75th percentiles, whiskers = min and max.

Regarding visual outcomes, JOAG had the highest proportion of eyes with VA ≤ 0.5 at both baseline and final follow-up. In most subtypes, there was no significant change in logMAR VA between baseline and final follow-up. Complete case analysis (85 eyes) revealed significant VA worsening only in S_GNOA (*p* = 0.0251). A trend toward poorer VA outcome was also observed in S_GFCS (*p* = 0.18) and S_GAC (*p* = 0.19), although these did not reach statistical significance (Figure 5).

## 4. Discussion

Childhood glaucoma comprises a heterogeneous group of conditions with onsets ranging from infancy through adolescence, often resulting in significant long-term visual impairment. In this 10-year retrospective cohort from a tertiary pediatric referral center, secondary glaucoma accounted for the majority of diagnoses, with glaucoma following cataract surgery (S_GFCS) emerging as the most common subtype. This predominance is largely attributable to the number of patients who were followed many years after congenital cataract surgery, as well as other conditions associated with pediatric glaucoma. This trend aligns with reports with higher frequencies of secondary glaucoma in similar populations [10,13]. Our findings are consistent with prior reports from North American cohorts that similarly identify secondary glaucomas—particularly post-cataract surgery glaucoma—as the dominant pediatric glaucoma subtype, in contrast to data from many developing regions where PCG predominates [18,19,20].

The distribution of pediatric glaucoma subtypes varies substantially across geographic regions, likely influenced by genetic, socioeconomic, and healthcare system factors [12,13,14,20,21,22]. Studies from Asia, Africa, and parts of South America consistently report higher proportions of PCG, often attributed to increased consanguinity, founder mutations, and delayed access to early surgical intervention [23,24,25,26,27]. In contrast, Western populations benefit from improved neonatal screening, earlier diagnosis, and advances in congenital cataract management, which may partially explain the lower prevalence of PCG and higher representation of secondary glaucomas observed in our cohort.

Surgical intervention patterns in our cohort reflected disease severity and underlying mechanisms. Primary glaucomas (PCG and JOAG) and S_GNSD demonstrated the highest surgical burden, consistent with their frequently more refractory IOP profiles and congenital anomalies of the trabecular meshwork or outflow system. Although JOAG is sometimes considered analogous to adult-onset open-angle glaucoma, a substantial proportion of JOAG eyes in this cohort required incisional surgery, underscoring the potential aggressiveness of the disease in pediatric and adolescent patients [28]. Nonetheless, selected JOAG cases were successfully managed medically or with laser therapy, reflecting heterogeneity in disease severity and progression.

In contrast, surgical decision-making for glaucoma associated with acquired conditions (S_GAC) was more individualized. In cases where visual prognosis was limited by trauma, advanced amblyopia, or concurrent ocular pathology, conservative medical management was often favored over aggressive surgical intervention [29]. This pragmatic approach reflects the necessity of weighing IOP control against overall visual potential and quality of life in pediatric patients.

While meaningful reductions in IOP were achieved across nearly all glaucoma subtypes, these improvements did not consistently translate into better visual outcomes. This dissociation highlights the multifactorial nature of visual impairment in childhood glaucoma. Worse baseline visual acuity and higher presenting IOP were strongly associated with poorer final vision, suggesting that many patients had already sustained irreversible optic nerve or visual pathway damage by the time of diagnosis. These findings emphasize that IOP control alone, although essential, is often insufficient to restore or preserve vision once structural or developmental damage has occurred.

Visual outcomes varied considerably among subtypes. Glaucoma associated with non-acquired ocular anomalies (S_GNOA) demonstrated a statistically significant decline in visual acuity over time. Given that these eyes frequently exhibit anterior segment dysgenesis or other congenital structural abnormalities, this progressive visual deterioration likely reflects intrinsic anatomic limitations rather than inadequate IOP management. These results underscore the importance of counseling families regarding guarded visual prognosis, even when acceptable pressure control is achieved.

The poor visual outcomes observed in the JOAG subgroup warrant particular consideration. Although JOAG typically presents after visual system maturation, many patients in this cohort were referred late in the disease course, often after prolonged periods of uncontrolled IOP. High presenting IOP, advanced optic nerve cupping, delayed recognition due to subtle early symptoms, and inconsistent access to ophthalmic care likely contributed to irreversible visual damage before treatment initiation. These findings highlight that JOAG cannot be assumed to follow a benign course and reinforce the necessity of early detection and sustained monitoring, even in older children and adolescents.

Importantly, visual outcomes in pediatric glaucoma cannot be attributed solely to glaucomatous optic neuropathy. Amblyopia, significant refractive error, nystagmus, corneal opacity and coexisting ocular or systemic anomalies frequently influence final visual potential. Although comprehensive pediatric ophthalmic and amblyopia care was provided to patients in this cohort, the retrospective nature of the study limited our ability to quantify the independent contribution of these factors. Prospective evaluations incorporating standardized visual assessments and detailed amblyopia management data would help clarify their relative impact.

Differences in follow-up duration across glaucoma subtypes may have influenced both visual and IOP outcomes, particularly in subgroups with shorter observation periods such as PCG. However, the absence of substantial late IOP rebound or visual improvement in subtypes with longer follow-up suggests that baseline disease severity and inherent visual potential, rather than follow-up duration alone, were the primary drivers of outcome differences. Nonetheless, this limitation is inherent to retrospective cohort studies and should be considered when interpreting comparative results.

The strengths of this study include a decade-long follow-up period, subspecialty management within a dedicated pediatric glaucoma program, and use of standardized CGRN classification to ensure consistent subtype categorization. Several limitations must be acknowledged, including the retrospective study design, modest sample sizes within certain subgroups, incomplete quantitative visual acuity data in very young children, and the presence of coexisting ocular pathology that complicates attribution of visual outcomes exclusively to glaucoma. Additionally, the relatively small number of PCG cases in this cohort limits the generalizability of subtype-specific analyses and raises the possibility of sampling bias. This likely reflects regional referral patterns and early surgical management outside our institution rather than true disease prevalence. Consequently, PCG-specific visual and surgical outcomes should be interpreted cautiously.

## 5. Conclusions

In this 10-year retrospective study, secondary glaucoma accounted for the majority of pediatric cases, with glaucoma following cataract surgery emerging as the most common subtype. Although meaningful reductions in intraocular pressure were achieved across nearly all glaucoma categories, visual outcomes frequently remained limited. Subtypes such as S_GAC and S_GNOA were associated with particularly poor visual prognoses, reflecting the influence of coexisting structural abnormalities, trauma, inflammation, or congenital anomalies.

These findings highlight the critical importance of early identification, comprehensive multidisciplinary care, and individualized management strategies for children with glaucoma. Improving long-term visual outcomes will require not only effective IOP control but also timely amblyopia management, precise refractive correction, and careful monitoring of associated ocular or systemic conditions. Continued research, including prospective, standardized studies, is needed to further refine treatment paradigms and support better visual prognoses in this vulnerable population.

## Figures and Tables

**Table 1:** Demographic and clinical characteristics of the study cohort, including distribution of glaucoma subtypes, number of eyes affected, sex, race, age at first and last visits, duration of follow-up, and laterality.

Characteristics	PCG	JOAG	S_GAC	S_GNOA	S_GNSD	S_GFCS	GS	Total	*p* Value
Number of patients, n (%)	7 (6.7)	19 (18.1)	21 (20)	7 (6.7)	16 (15.3)	24 (22.9)	11 (10.5)	105 (100)	-
Number of eyes, n (%)	9 (5.4)	37 (22)	26 (15.5)	10 (6.0)	27 (16.1)	39 (23.2)	20 (11.9)	168 (100)	-
Gender (patients, n)									0.748
Male, n (%)	4 (51.7)	9 (47.4)	16 (76.2)	2 (28.6)	9 (56.3)	12 (50)	4 (36.4)	56 (53.3)	0.956
Female, n (%)	3 (42.8)	10 (52.6)	5 (23.8)	5 (71.4)	7 (43.7)	12 (50)	7 (63.6)	49 (46.7)	0.743
Race, Caucasian, n (%)	5 (1.0)	14 (13.3)	11 (10.5)	5 (1.0)	11 (10.5)	17 (16.2)	9 (8.57)	72 (68.57)	-
Age at first visit (year) *	2.98 (1.91)	8.24 (7.07)	7.9 (4.95)	3.25 (4.7)	3.9 (3.36)	3.19 (5.37)	6.8 (4.39)	5.52 (5.6)	-
Age at last visit (year) *	4.26 (1.96)	14.1 (7.83)	12.8 (4.23)	9.06 (3.28)	9.17 (5.3)	10.08 (5.48)	12 (3.89)	10.93 (5.9)	-
Duration follow-up (year) *	1.29 (1.71)	5.86 (3.87)	4.78 (3.05)	5.81 (3.72)	5.27 (3.39)	6.89 (2.67)	5.2 (3.23)	5.41 (3.37)	0.066
Laterality (patients)									0.109
Bilateral, n (%)	2 (28.6)	18 (94.7)	5 (23.8)	3 (42.9)	11 (68.8)	15 (62.5)	9 (81.8)	63 (60)	0.481
Unilateral, n (%)	5 (71.4)	1 (5.3)	16 (71.2)	4 (57.1)	5 (31.2)	9 (37.5)	2 (11.2)	42 (40)	0.361

* Data shown in mean (SD). Abbreviations: PCG, primary congenital glaucoma; JOAG, juvenile open-angle glaucoma; S_GAC, secondary associated with acquired conditions; S_GNOA, secondary glaucoma associated with non-acquired ocular anomalies; S_GNSD, secondary glaucoma associated with non-acquired systemic/syndromic disease; S_GFCS, secondary glaucoma following cataract surgery; GS, glaucoma suspects.

**Table 2:** Baseline intraocular pressure (IOP), cup-to-disk ratio (CDR), and spherical equivalent refractive error for each glaucoma subtype. Values are presented as mean ± standard deviation for IOP and CDR, and median (IQR) for refractive error.

Glaucoma Subtypes	IOP (mmHg)		Cup-to-Disk Ratio (CDR)			Spherical Equivalent	
	(Mean ± SD)	Eyes (n)	(Mean ± SD)	Eyes (n)	*p*-Value	(Median, IQR)	Eyes (n)
PCG	32.89 ± 14.31	(n = 9)	0.64 ± 0.25	(n = 8)	0.01 ‡	−2.87, 4.5	(n = 9)
JOAG	27.16 ± 10.41	(n = 37)	0.61 ± 0.19	(n = 30)	0.01 ‡	−4.25, 6.5	(n = 37)
S_GAC	25.17 ± 11.56	(n = 18)	0.38 ± 0.14	(n = 23)	0.008 †	−0.50, 4.5	(n = 26)
S_GNOA	17.81 ± 4.61	(n = 10)	0.28 ± 0.17	(n = 7)	-	+0.5, 13.87	(n = 10)
S_GNSD	26.87 ± 12.62	(n = 23)	0.53 ± 0.25	(n = 21)	0.001 †	−0.75, 4.87	(n = 27)
S_GFCS	20.47 ± 7.72	(n = 19)	0.37 ± 0.22	(n = 31)	0.007 †	+9.63, 8.87	(n = 39)
GS	17.22 ± 3.71	(n = 15)	0.60 ± 0.21	(n = 16)	-	+1.0, 2.75	(n = 20)
Overall	24.41 ± 10.85	(n = 131)	0.46 ± 0.23	(n = 136)	-	−0.63, 4.25	(n = 168)

‡ Two-tailed Student’s *t*-test between two groups of primary and secondary glaucomas. † Two-tailed Student’s *t*-test between two groups of secondary subtypes.

**Table 3:** Baseline visual acuity (VA), categorized by unilateral and bilateral glaucoma, comparing glaucomatous and fellow eyes. Qualitative VA categories (e.g., CSM, CF/HM, LP, NLP) are shown for patients unable to perform quantitative testing. Eyes from 23 patients too young for quantifiable testing were excluded.

BCVA (LogMAR)	Unilateral Glaucoma		Bilateral Glaucoma		
	Glaucomatous Eye n (%)	Non-Glaucomatous Eye n (%)	Better Seeing Eye n (%)	Worse Seeing Eye n (%)	Eyes Equal n (%)
≤0.3	5 (4.2)	14 (11.7)	4 (3.3)	3 (2.5)	16 (13.3)
>0.3–≤0.6	1 (0.8)	1 (0.8)	4 (3.3)	2 (1.6)	4 (3.3)
>0.6–≤1.0	0	0	1 (0.8)	3 (2.5)	2 (1.6)
>1.0	0	0	0	1 (0.8)	0
CSM	2 (1.6)	5 (4.2)	2 (1.6)	0	8 (6.7)
CF or HM	6 (5.0)	1 (0.8)	1 (0.8)	1 (0.8)	0
LP + UFF	7 (5.8)	6 (5.0)	1 (0.8)	1 (0.8)	16 (13.3)
NLP	2 (1.6)	0	0	0	0

**Table 4:** Distribution of glaucoma surgeries performed across glaucoma subtypes, including trabeculectomy, trabeculotomy, goniotomy, Baerveldt implantation, combined procedures, and cyclodestructive laser treatments.

Surgery Procedure	PCG	JOAG	S_GAC	S_GNOA	S_GNSD	S_GFCS
	* n = 8 Eyes	n = 27 Eyes	n = 6 Eyes	n = 4 Eyes	n = 20 Eyes	n = 20 Eyes
Trabeculectomy	1	10	2	1	9	3
Trabeculotomy	1	2	0	0	0	0
Trabeculectomy + Baerveldt	0	4	0	0	4	6
Baerveldt implantation	2	6	1	1	0	4
Goniotomy	3	1	0	0	4	1
Iridectomy	0	0	2	1	1	1
Cyclodestructive laser	0	2	0	1	0	4
Baerveldt + Iridex laser	1	2	1	0	2	5

* Values represent number of eyes undergoing each procedure; eyes may be represented in more than one category if they received multiple interventions over time.

## Data Availability

The datasets generated and/or analyzed during the current study are available from the corresponding author on reasonable request.

## Abbreviations

The following abbreviations are used in the manuscript:

IOP	Intraocular pressure
CGRN	Childhood Glaucoma Research Network
BCVA	Best corrected visual acuity
PCG	Primary congenital glaucoma
JOAG	Juvenile open-angle glaucoma
S_GAC	Secondary associated with acquired conditions
S_GNOA	Secondary glaucoma associated with non-acquired ocular anomalies
S_GNSD	Secondary glaucoma associated with non-acquired systemic/syndromic disease
S_GFCS	Secondary glaucoma following cataract surgery
GS	Glaucoma suspects
CSM	Center, steady, maintained
CNSM	Center, not steady, maintained
CSNM	Center, steady, not maintained
NCSM	Not center, steady, maintained
CF or HM	Count finger or hand motion
LP	Light perception
NLP	No light perception
UFF	Unable to fix and follow
